# Empowerment of Lay Mental Health Workers and Junior Psychologists Online in a Task-Shared, Rural Setting in Kerala, India

**DOI:** 10.34172/ijhpm.2024.7566

**Published:** 2024-06-12

**Authors:** Rekha Pallikkuth, T. Manoj Kumar, Claudia T. Dictus, Joske F. G. Bunders

**Affiliations:** ^1^Department of Clinical Psychology, Mental Health Action Trust, Calicut, India.; ^2^Athena Institute, VU University Amsterdam, Amsterdam, The Netherlands.; ^3^Mental Health Action Trust, Calicut, India.

**Keywords:** Supervision, Empowerment, Community

## Abstract

**Background:** Patients with severe mental health issues who live in isolated rural areas are difficult to reach and treat. Providing effective treatment is difficult because mental health problems are complex and require specialized knowledge from a range of professionals. Task-sharing with lay mental health workers (LMHWs) has potential but requires proper training and supervision to be effective. This article reports on the challenges and facilitators experienced in empowering LMHWs in their role, with the help of a technology supported supervision group. The study sought to understand the functioning of the Empowering Supervisory Group (ESG) in the context of junior psychologists and LMHWs in rural India, and investigate how they experienced it by exploring challenges, lessons and empowerment.

**Methods:** Qualitative analysis of interviews with the 22 ESG participants and their supervisors.

**Results:** A total of three discrete phases of supervision were identified where supervisors responded to the changing needs of the group. This began with building trust at a baseline level, tackling issues with competence and autonomy and finally experiencing meaning and impact through self-determination. The experience of empowerment even in an online setting was very beneficial given the challenges of working in rural areas.

**Conclusion:** Empowerment based supervision of LMHWs and junior psychologists online enables a level of engagement that positions them to engage in community mental health practices with greater independence and confidence.

## Background

**Key Messages**

**Implications for policy makers**

- Lay mental health workers (LMHWs) are a viable addition to mental health systems in low- and middle-income countries (LMICs), provided appropriate training and supervision is available.
- The potential of online services to increase access and close the mental health treatment gap can also be extended and to creating effective structures for the support of LMHWs or less experienced staff.
- With more holistic approaches to mental health, systems of care need to adjust to meet the needs of patients and practitioners. This paper demonstrates how empowerment-based supervision can support care practitioners in navigating the complexity of such approaches.

**Implications for the public**

Meeting mental health needs in low- and middle-income countries (LMICs) or resource scarce settings has proven very challenging and requires innovative strategies to address. The potential of online group supervision for junior staff and lay mental health workers (LMHWs) to improve the quality of care and strengthen confidence in providing complex care was explored in this study. It was found that this form of training improved outcomes, but that in order for it to be effective supervisors had to be flexible and attentive to the changing needs of their supervisees.

It is well established that the global treatment gap in mental health is most pronounced in low- and middle-income countries (LMICs) due to a shortage of qualified staff.^1,2^ Currently, there are only 1.93 trained mental health professionals for every 100 000 people in India, compared to 71.7 professionals per 100 000 people in many high-income countries.^3,4^ Furthermore, high levels of stigma present a particular issue given the experiences of discrimination which can further exacerbate mental health symptoms.^4,5^ This is one example of the type of complexity involved in this field that has resulted in theoretical shifts towards more holistic, well-being–oriented approaches to treatment.^6-8^ However, such theoretical shifts may also exacerbate the staff shortages as pervasive measures such as the training of lay mental health workers (LMHWs) for task-sharing may be ill-equipped to address mental health at a systems level.^9^ LMHWs are defined as individuals with no previous professional mental health training or background who are employed to help treat and manage common mental health disorders where their potential in LMIC settings like India through the application of contextualized knowledge has been well established.^9,10^ For instance, LMHWs typically come from the communities they serve and therefore have higher acceptability which may contribute to the reduction of stigma.^9^ Furthermore, studies involving LMHWs in LMICs have shown great potential for LMHWs’ effectiveness on mental health outcomes in India in particular.^11,12^

 As such, organizations aiming to address the treatment gap such as the Mental Health Action Trust (MHAT), an organization that provides free mental healthcare to underserved populations require approaches that build on this potential in a way that accommodates evolving practices and complexity. This study aims to explore such an approach through the unique combination of empowerment-based supervision and e-health technology. Although the efficacy of LMHWs in low-resource settings through well-designed training programmes has been established, common approaches are also found to be insufficiently supportive when it comes to building confidence and competences required for LMHWs to act independently in the dynamic environment of psychosocial interventions.^13^ Research indicates the need for ongoing training, supervision and mentorship in general to encourage continual learning and development.^12-14^ Furthermore, the psychological empowerment of staff more generally has been associated with better outcomes for patients.^15^ It follows that empowerment-based supervision of LMHWs could enable them to provide better interventions that address the complexity of well-being–oriented care. However, persistent resource and geographic limitations apply as much to supervision as they do to treatment, such that it is necessary to identify cost-effective means of empowering LMHWs to carry out independent psychosocial interventions. Changes in practice, in part arising from the COVID-19 lockdowns, have resulted in increased consideration of digital practices as tools for supervision and mentorship.^12,16-19^

 With the political, social and historical shifts within systems of care moving away from linear structures and towards synergistic interconnected approaches and multidisciplinary teams, different ways of orienting are required on the part of staff.^17,20,21^ In MHAT, groups of care providers make up a multidisciplinary team including mental health professionals, non-professionals and community volunteers; providers of housing, employment services, education and training, and related support services; as well as families and carers.^21,22,23^ In parallel to the gradual trend towards holistic care, theory and research in the area of job performance have increasingly highlighted the importance of psychological empowerment as a key determinant of success and resilience.^24,25^ Psychological empowerment is understood as the degree of intrinsic motivation or self-efficacy of an employee on the basis of experiencing meaning in their work, self-perceived competence, the sense of self-determination or an ability to make decisions and the perceived impact of their work.^26-28^ While multiple frameworks for supervision generally and clinical supervision exist, for instance Proctor’s three function model, most are intended for one-to-one settings and classical care structures.^29,30^ The concept of psychological empowerment as outlined in Figure has been used for the particular complexity of community mental health settings, and has been applied in the context of both leadership and supervision in numerous quantitative studies and guidelines.^27^ Though this has shown that the key aspects of meaning, impact, self-determination and competence to have a significant effect on outcomes, such as work engagement and meaningful work experience, until recently none had qualitatively traced the ways in which these aspects can be targeted and strengthened in leadership.^27,29^ Based on the findings of Bunders et al, this study used understandings of leadership skills, context-specific tools and problem-solving approaches to structure the supervision of LMHWs in order to enable organization wide capacities for change and navigating complexity.^27^

**Figure:**
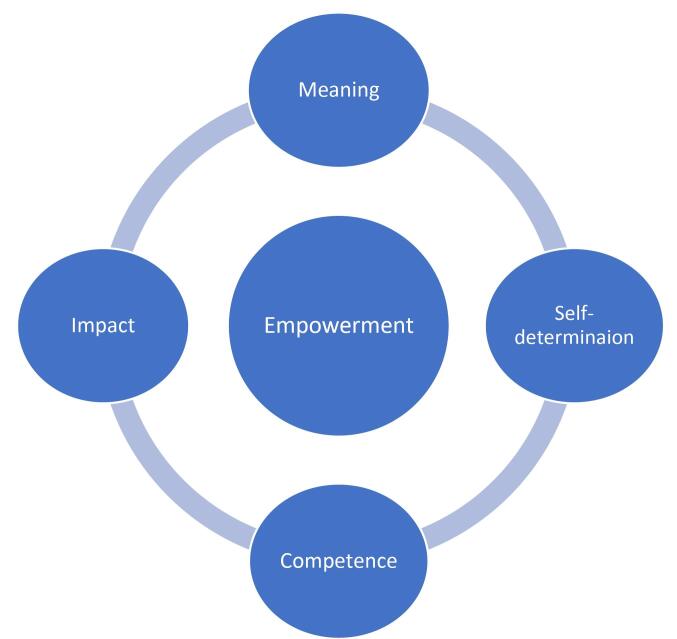
Aspects of Psychological Empowerment, Based on Bunders et al.^28^

 Concerning the resource constraints in providing supervision, the shift in practices encouraged by the COVID-19 pandemic would suggest the exploration of the potential of online supervision. Though the potential of telemedicine and other digital practices is well established by the World Health Organization (WHO) and government of India in regards to furthering the health of individuals and communities, some potential barriers have also been identified.^31,32^ For instance, poor internet access or a low bandwidth; limited familiarity with or openness to online mental health services; scheduling across time zones; and the loss of non-verbal communication that typifies in-person interactions have been identified as potential challenges.^31-33^ As such, practical studies that evaluate the feasibility of online solutions and explore how actors navigate these challenges are needed.

 In its exploration of the potential for online supervision to strengthen psychological empowerment in LMHWs and junior staff to enable them to navigate the complexity of current approaches to community mental healthcare, this study has established the following research questions:

1. What does online supervision look like in the context of LMHW and junior psychologist supervision in rural India?
2. How do junior psychologists and LMHWs experience online supervision?
  - What challenges do they experience?
  - What can be learned from their challenges?
  - What benefits did they experience, and can these be understood within the framing of empowerment?

## Methods

 This article explores the set-up, running and evaluation of an online supervision group for community clinics in India, as well as the challenges and opportunities that emerge. The MHAT developed its tele-psychiatry unit in 2014 for the provision of pharmacological interventions to patients with severe mental illness in rural clinics. The supervision program considered in this article provided an extension of these practices and aimed to provide supervision to both junior psychologists and LMHWs to empower them to meet various functions, especially pharmacological interventions to those with severe mental illnesses in community settings. Ongoing training and supervision by experienced clinical psychologists essential for the junior psychologists and LMHWs who manage the individual community clinics. The idea that each community clinic could benefit from the Empowering Supervisory Group (ESG) arose from discussion among junior psychologists and LMHWs. It employed a qualitative triangulation approach to understand participant’s perceptions regarding the intervention, what challenges they encounter, and what aspects of empowerment were experienced (including increased impact, meaning, self-determination and competence).

###  The Study Context: MHAT Community Clinics in Kerela, India

 The current study was carried out in the context of the MHAT, a non-government organization based in Kozhikode, in the Indian state of Kerala. MHAT provides free mental health services to economically disadvantaged people in several districts of Kerala. Comprehensive multidisciplinary care has been provided by LMHWs through local partnership with the wider health system since 2009. The LMHW share the bulk of community-based work, and tele-psychiatry units are a key element of this model. The roles of LMHWs range from screening and regular domiciliary monitoring of patients to providing group and individual psycho-social interventions, rehabilitation, and family-focused interventions. The tele-psychiatry unit has played a crucial role in pharmacological interventions since 2013 and, as covered in this study extended into supervising psycho-social interventions from May 2021. The study was conducted from October 2021in the transition from the COVID-19 pandemic.

 Prior to this study, the primary function of the tele-psychiatry unit was related to pharmacological management through tele-consultation with psychiatrists, generation of e-prescriptions and supervision of pharmacological management for psychologists and psychiatric social workers who conduct the regular face-to-face follow-up of patients.

 In this study, a trained clinical psychologist offered group sessions of clinical supervision on both group-based and individual psycho-social interventions over a period of six months (May–October 2021). The ESG conducted a regular weekly session for each clinic team member, including LMHWs, junior psychologists and the supervisor. A total of 22 clinic teams joined the discussions each week. Each ESG session lasted for up to an hour, and started with reviewing the previous session’s action plans. The discussions then focused on out-patients and their care plans. Challenges and new lessons were shared in the group. The sessions focused on improving the quality of clinical work practices and understanding the decision-making processes of LMHWs and psychologists and their impact on patients, multidisciplinary teams, and rural community clinics in the Wayanad, Malappuram, Kozhikkode, and Palakkad districts of Kerala. The supervisors kept written records and participants were encouraged to do the same, along with recorded group supervision sessions via Zoom.

###  Study Design and Instruments

 A total of 17 people participated in the ESG group and thus the study. The data collection instruments included observational notes from the supervisors, socio-demographic questionnaires from the participants, semi-structured interviews with the participants, and evaluation forms completed by the participants after the conclusion of the supervision program. This form of triangulation, with multiple forms of data collection is known to increase the validity of qualitative findings. The research team consisted of research-trained mental-health workers employed at MHAT including a psychiatrist, a clinical psychologist, and three general psychologists as well as a senior researcher in innovation studies outside of the organization. The interviews were designed within the research team on the basis of the aspects of psychological empowerment with specific prompts but also space for emergent responses and discussions. Prompts included questions about the process of group supervision and the skills required for providing psychosocial interventions as well as potential challenges or issues experienced during supervision. This study applied a social constructivist epistemological framework in that it regards human learning as constructed through social interaction as a shared (rather than individual) experience.^34^

###  Recruitment Process 

 Participants for the ESG group were recruited using purposive sampling, where all eligible junior psychologists and LMHWs were invited to join over the phone and reminded twice. Participants were assured that their involvement was voluntary and that they could withdraw from the study with no negative effects on their employment. Online supervision was offered to 12 LMHWs and four psychologists by a clinical psychologist supervisor. Each participant received at least 12 sessions of an hour each over a six-month period. The supervisor had over 12 years’ experience in task-shared, recovery-oriented community mental health services.

###  Analysis

 Interviews were transcribed verbatim omitting personal identifiers and the transcripts were sent to participants for a member check to ensure validity. The information from the demographic questionnaires was collated into tables of participant characteristics and evaluation sheets and supervisory notes were anonymized for analysis. The interviews were analysed using deductive content analysis with inducive shifting, where the codes were made up of predetermined categories relating to psychological empowerment and the interview questions but accommodation was made for consistently occurring emergent codes. This process was led by three psychologists with research training who cross-checked each-others coding and verified the significance of emergent codes. Coding was conducted in Microsoft Word and an analysis matrix was created in Excel with five columns (research question, main theme, sub-theme, code, and interview excerpts) which was discussed within the full research team. These findings were supplemented with comments emerging from the evaluation sheets and supervisor’s notes. Three phases of experience within the supervisory process were identified relating to increasing and were then used to further structure the analysis as shown in the results below. Finally, the research findings were discussed with non-MHAT research colleagues for peer debriefing and feedback.

## Results

 As mentioned before, this study focused on understanding the impact of the ESG on participants; the challenges experienced, but also aspects of empowerment. In Table 1 the characteristics of participants are shared. There were four psychologists, with an average of two years of experience, plus 12 lay workers, with an average of four years’ experience. Finally, there was one specialised psychologist present with 12 years of experience. The experiences of supervision can be loosely categorized into three phases on the basis of the learning process, and the data is presented per phase as summarized in Table 2. In phase 1, lack of familiarity with the process and implementation concerns caused initial apprehension and confidentiality concerns. This was remedied among other things by supporting participants to lobby their administrative teams to set aside specific time for ESG, which was experienced as empowering and increased ownership of the group. With improvements leading into the second phase, it was possible to focus on development in the four dimensions of psychological empowerment for instance through emotional support and stress reduction techniques. In the final phase, participants were fully in control of decision-making and were confident to share cases and learn from each other. We discuss the findings in terms of these three phases below.

**Table 1:** Characteristics of Participants

** Postgraduate-Level Psychologist (Supervisee)**		**LMHW (Supervisee)**		**Specialized Clinical Psychologist **	
**Number **	**Average Years’ Experience**	**Number **	**Average Years’ Experience**	**Number **	**Average Years’ Experience in Task-Shared, Community Mental Health Setting**
4	2	12	4	1	12

Abbreviation: LMHW, lay mental health worker.

**Table 2:** Challenges and Lessons Learned in Each Phase

	**Phase 1**	**Phase 2**	**Phase 3**
Competence	Fear to be found incompetent increased insecurity Competence in use of online applications	Assessing strengths of participants to encourage problem solving Discussing cases to work on clinical values, relationships, ethical practice etc Recorded zoom sessions as a guide/reference Targeted feedback	Awareness of competences and needs- tackling specific challenges with documentation and administrative policies Sharing experiences and providing corrections for each other
Autonomy (self-determination)	Ask for input and provide opportunities for decision-making Encouraging independent action on time and space for supervision	Encouraging boundary setting and clinical problem solving Recording sessions to allow the choice to listen at any time	Ownership and decision-making about ESG Independent action on documentation and administration Sharing of own success stories
Impact	Verbal reassurance Positive feedback	Opportunity to present successes Encouraged to give each other positive feedback	Encourage supervisees to take new responsibilities roles
Meaning	-	Rationale of each activity and expected outcomes should be discussed	Provide opportunity to share success stories

Abbreviation: ESG, Empowering Supervisory Group.

###  Phase 1

 The issues raised here can be summarized as: lack of competence, self-determination (autonomy), and impact. These issues were addressed by encouraging participants’ self-determination, collective decision-making and providing a sense of their impact.

####  Lack of Competence

 Competence, understood as the ability to work at the level outlined in the description of their position, proved a challenge given the significant differences in educational background and work experience. Participants had diverse expertise, but also had different gaps in skillsets for treating mental illnesses. To negotiate this, the first phase involved a process of taking stock of these differences through supervisor’s enquiries, but also an emphasis on strengths rather than gaps to accommodate pervasive insecurity and lack of trust. For instance, participants expressed serious concerns about confidentiality which could be understood both as a lack of trust in their own abilities, and a potential fear of being seen as incompetent. This impacted attendance and participation as well;

 “*Initially, I was not confident in that group. Someone is monitoring my work closely and discussing was making me more anxious…I thought about skipping the sessions*” (LMHW, group 3).

 “*Lack of comfort to engage with ESG was there in me in the initial days…I had fear that whether my points are scientific or not, ethical or not…etc”* (P2).

 Therefore, the supervisor directed their enquiry towards examples of participants’ struggles, and encouraging them to identify the skills and strengths that lead them to particular solutions. This was supplemented by establishing a strong supervisor–supervisee relationship through individual phone conversations; providing positive feedback on their participation and clarifying expectations, rules and standards.

 “*Initially, my supervisor was calling me after the ESG sessions and enquired about my comfort level and she sought feedback for herself to improve her next session. She was actively showing interest in my participation and encouraging me to contribute more. That gave me confidence to her inquiries in the sessions*” (LMHW, group 3).

 “*She described…core values and guiding principles in our service which helped us to clarify basic ethical concerns”* (P1).

####  Autonomy

 A sense of autonomy (self-determination) is necessary in order to navigate community mental health work, without which nobody will take responsibility for decision-making. Autonomy involves making active choices based on individual needs and thoughts, to which two key issues in phase one relates: first, participants struggled to agree on a weekly slot for the ESG; and second, they were unable to resolve practical issues. Participants indicated challenges agreeing on a time because of differences in availability and obtaining permission to allocate hours to ESG, which was exacerbated by understaffing and competing work demands. This lack of control over time planning and the resistance to take agency in decision-making was noted as a barrier by the supervisor. Similarly, connectivity issues and interruptions were difficult to resolve in initial sessions.

 “*During most of the sessions, our group members go through the issue of connectivity or sometimes issues with external distractions or privacy…” *(LMHW, group 7).

 In contrast to traditional supervision where participants experience a controlled environment, in the simple sense that a closed space is selected, participants experienced the online setting as a risk to their privacy as others might overhear their sessions. Thus, lack of autonomy was also seen in the inability to create a practical space to engage in ESG.

 These issues were remedied by supervisors encouraging participants to take stock of their needs and independently decide on a course of action, as noted in the quote:

 “*Our supervisor encouraged us to present our issue of finding a time for ESG in [the] administrative level. At last, we were allowed to get protected time for supervision”* (LMHW, group 5).

####  Impact

 Impact, or understanding one’s own role and importance in an organization, is crucial to working as LMHWs and psychologists. Strongly linked to the challenges of competence, differences in experience and education lead to a lack of confidence as participants were unsure of their comparative importance. This manifested itself as interpersonal issues as some felt too unimportant to voice their opinions, or felt threatened or offended by the expression of others:

 “*I felt difficulty when one group member self-boosts her activities. I keep silent when she is in group*” (LMHW, group 8).

 This was also tied to issues of seniority and authority. Passive aggression was continually cited within the workgroups, and particular forms of this arose because of the context. For instance, connection issues were used as an excuse to leave the group as a form of passive aggression, so that even genuine connection issues were sometimes interpreted in this way.

 “*Sometimes members faced difficulties in tolerating uncertainties of someone’s sudden silence or leaving”* (Supervisor).

 “*One group member was dominating the group several times and others were not getting time [to]express their opinions. I felt bad. I suddenly left the group”* (LMHW, group 6).

 Thus, tension arose around individuals leaving without warning, not using video or not speaking. There were also occasional instances of explicit conflict:

 “*One of my dominating colleagues questioned my credibility in group. I felt very bad in that. I have not left the meeting” *(LMHW, group 5).

 Some explanation for these insecurities might also be provided by a lack of clarity about the distribution of tasks and roles within MHAT, leading to ambiguity and conflict as in the example below.

 “*Most of the time, [the] psychologist is interfering my work. When we discuss cases in ESG, she presented my roles as her roles”* (LMHW, group 4).

 These issues were addressed through direct conversations with relevant group members, for instance by addressing those dominating the group discussion, or encouraging those who were reluctant. In some cases, an upper limit for the degree of input was established so that the supervisor could give reminders or interrupt when it was exceeded.

 “*Our supervisor prompted to limit the discussion of members who tried to dominate the ESG*” (P3).

 The issues arising from uncertainty in task-shared roles were further resolved through individual discussions with the relevant parties.

 “*[The] supervisor explained my roles specifically, to me those specific tasks helped me to clear my roles”* (P5).

###  Phase 2 

 Having established a baseline of trust and respect, the second phase of ESG began in which different challenges and strategies were seen and worked with. With the increased confidence, participants began to participant more in discussions and decision-making, which results in a greater sense of meaning and impact. Further, having established key strengths in relation to competences in the first phase, it was possible to move towards evaluating gaps and addressing problems. Though each of these aspects (competence, self-determination, and impact) were closely intertwined, data was extracted for each separately.

####  Building Competence

 Competence in phase two was primarily related to achieving a complete baseline set of skills and knowledge appropriate to participants’ position and education by identifying and accommodating particular gaps. For instance, competences related to clinical values, client relationships, ethical value-based practice, appreciation of diversity and evidence-based practice. In each session, the supervisor focused on particular clinical activities (eg, patient follow-up, psychosocial assessments, etc) and review participants’ case notes and documentation to facilitate enquiry and reflective practice. In doing so, key challenges related to knowledge, skills and attitudes of participants were identified.

 Some examples of a lack of knowledge related to psychosocial assessments, interventions and ethical values, came up. Having created a trusting environment, it was easier for participants to open up, and it became clear that some junior psychologists lacked basic knowledge about psychosocial assessments and interventions, and were therefore not confident to comment on or support their LMHWs interventions in the community. Both junior psychologists and LMHWs struggled with the concept of a therapeutic relationship and building rapport with patients, and typically launched into interventions without preamble. Similar disregard was given to the concept of continuity of care, which presents a significant ethical issue.

 “*I was anxious in taking lead role in care planning because of my lesser experience in clinical setting. I have to prepare for each outpatient care”* (P1).

 “*Each patient in the next outpatient service were discussed in our Zoom sessions. I was not aware of steps and rationale in each decision-making process of patients. I don’t have enough knowledge on scientific interventions for [a] person with severe mental illness in community” *(P3).

 Further developing competences was particularly challenging in some cases given individual difficulties in grasping abstract concepts or difficulties maintaining attention for extended periods of time.

 “*Some of the supervisees showed marked issues in their attention and comprehension*” (Supervisor).

 “*I take more time to understand some points…”* (P2).

 To address these issues, the supervisor took two approaches; building on the previous practice of focusing on strengths and emphasizing self-efficacy or the ability of participants to affect their own circumstances. This was done by encouraging members to share their success stories as well encouraging feedback and support.

*“[The] supervisor’s way of asking questions gave us different perspectives in psycho-social assessments and interventions. When we do proper assessments and interventions she gives as thumps ups. That is really a social reinforcement for us”* (P2).

*“The success stories from colleagues help me”* (LMHW, group 2).

 Participants cited three further factors that enabled their learning. First, attending in a comfortable space, which reduced the stress of travelling and allowed time for self-care. Second, participants reported that the recorded sessions helped to remind them of their assigned tasks and knowledge, which gave them a feeling of control over the previous session. Third, they reported it was a supportive environment in which they couldcommunicate their feelings openly.

*“Travelling all the way from home to Calicut for this discussion is not here. I can save 6 hours of travel time and utilize this time for more selfcare activities”* (LMHW, group 3).

*“Our supervisor appreciated the communications verbal and nonverbal…. She asked us to type the major points in chats. At the end of each session, major messages were shared in screen” *(P3).

 Finally, by focusing on strengths the supervisor helped participants to compensate for limitations and skills.

 “*My supervisor showed my specific skills in conducting group sessions” *(MHW, group 3).

*“It was interesting to find out their strength and reflect to them”* (Supervisor).

####  Autonomy

 Given the horizontal leadership of community healthcare, participants both experience the empowering leadership of others and were in turn leaders in their own setting. As such, autonomy and decision-making capacity are essential to their work, and though autonomy is a complex construct that includes a combination of skills and knowledge, the skills can be taught, measured, and most effectively developed through regular practice. Autonomy in phase two was strongly linked to competence where insecurity and a lack of guidance resulted in difficulty setting professional boundaries and engaging in clinical problem-solving.

 Issues in establishing professional boundaries were evident in the area of clinical decisions as some participants struggled with sharing information, fixing boundaries in the community, maintaining the division of responsibilities in clinics, and using time for clinical activities:

 “*I have confusion in sharing some confidential matters of patients in [the] community clinic volunteer group, especially sexual abuse issues*” (LMHW, group 2).

*“I had difficulty in stopping unnecessary gossiping from community clinics, especially community clinic staff members’ internal politics” *(LMHW, group 3).

*“Sometimes, volunteers interfere in clinical activities like home visits. They will have [the] tendency to intrude into discussions [during] home visits” *(P4).

 Participants also identified issues related to clinical problem-solving, which in this context involves understanding symptoms and identifying and prioritizing potential diagnoses. These actions in turn require an investigative mindset, collecting and processing information, evaluating and analysing this information, and setting actionable goals. Struggling with these competencies resulted in indecisiveness and confusion. Participants gave examples relating to care plans and implementation.

*“I am not confident to communicate in group about my care plan”* (LMHW, group 4).

*“I think we need to be clearer in rationale and scientific evidence about interventions and principles”* (P3).

 To tackle these problems, supervisors focused on providing opportunities to practice decision-making, for instance by asking for input as a starting point in the discussion of clinical activities, requiring increasing participation in decisions in different domains, and discussing possibilities and the limitations and risks associated with them. Collaborative decision-making was encouraged and a protocol developed that entailed listing alternative relevant actions, identifying their possible consequences, assessing the probability of each consequence occurring, establishing the relative importance of each consequence and integrating these probabilities to identify the most attractive course of action and associated achievable goals.

####  Impact and Meaning

 The improvement in autonomy and competence directly affected the participants’ sense of impact and meaning. By more thoroughly understanding and implementing MHAT’s protocols in relation to daily activities such as outpatient services, vocational rehabilitation, support groups and case management, participants were able to experience improvements in outcomes directly. However, some challenges were still experienced relating to a lack of knowledge on the rationale of their activities and not seeing the value of their daily practices to the organization. Concerning the former, participants often cited that they carried out certain activities without truly understanding why they were doing so. For instance:

 “*In Multiple Family Support Groups in my community clinics, I felt lack of confidence and stuck in continuing sessions. That caused absenteeism in participants*” (LMHW, group 2).

 The resulting impacts on the quality of the intervention likely furthered the sense that there was no meaning to the activity. According to the supervisor, this cycle contributed to problematic behaviours:

*“Recurrent unplanned leaves and absenteeism in meetings without any particular reasons were very evident their perception of unimportance”* (Supervisor).

 As such, the supervisor sought to break the cycle by explicitly discussing the mechanisms behind the different activities of MHAT in the ESG. These discussions were brought back throughout several sessions.

 “*In ESG, our supervisor asked the rationale of the activities before we do. She helped us to think the activities like Multiple Family Support Groups and Day care are helping others to improve others quality of life*” (LMHW, group 1).

###  Phase 3

 After four months, the nature of group sessions addressed a more managerial and organizational level of functions. After establishing a baseline of trust, and the second phase of targeted support, the dynamics created by empowerment-based leadership resulted in a shift in participants’ attitude through which they came to a sense of impact and meaningfulness that led them to take ownership of the overall functioning and success or failure of the organization. They felt empowered to take an active role in evaluating and addressing organizational challenges, and took part in decision-making. Participants took initiative in improving quality and standards in several organizational areas like policies and strategy planning. For this to be possible, significant developments in competence had to be made as supervisees needed to have capacities not only to act in the wide variety of situations arising in their own clinic, but to understand well enough to encounter commonalities and directions for improvement. This demonstrates a grasp of scientific knowledge and ethical and legal standards and policies as well as communicative and empathetic skills.

 Only one key competence still required addressing, which related to documentation. Documentation refers to the records an organization keeps and uses to inform decisions within community clinics. Participants demonstrated a lack of regard for this process which required further explanation of its significance, for instance relating to the reminders of organizational standards that could be found in documentation of MHAT. In ESG, they used Zoom to share screens to get feedback on group members’ best documentation models. Suggestions were gathered from group members and written on a Zoom collaborative whiteboard and saved for later use.

 “*I was using the presented documentations and suggestions as [a] reference point. When I want more clarity in this, I go through the recordings, which gives me more clarity”* (LMHW, group 9).

 Another particular form of documentation that was addressed related to task allocation, which is one of most important managerial roles in a task-shared community mental health setting. To strengthen this a common task platform was created to share tasks within supervision groups through their WhatsApp groups. This was shared via Zoom, and everyone could see exactly when their tasks were due and, more importantly, why. Thus, sharing tasks in the ESG Zoom session became transparent and well organized so that everyone knew exactly what we going on which made self-determination easier. Accountability for each member’s role in overall patient care might also to boost productivity.

*“Each ESG sessions started with screen sharing the task list. We will get [a] proper idea on which tasks are pending and explanation also needed. When you finish the task, you appreciate each other”* (P1).

 Finally, it was demonstrated in group interactions in phase three that participants felt more in control of their own behaviour (self-efficacy), able to take active decisions (autonomy) and thus felt like they could contribute (meaning/impact). For instance, they started to present their best practice models in all staff groups voluntarily. The achievement of impact and meaning empowered them, and they realized they could contribute something to their colleagues from their experience.

 “*Now I know my special skills in identifying community resources and proper utilization for our patients and family. Now I am confident to present this success stories in front of our other colleagues” *(P4).

## Discussion

 This research aimed to understand the potential of online supervision for improving the psychological empowerment of junior psychologists and LMHWs in rural India in order to enable them to navigate the complexity of psycho-social intervention approaches. The process, challenges and experiences of empowerment uncovered in the data analysis are discussed at length, followed by a brief discussion of technological aspects and limitations of the research and ending with a conclusion on this primary research aim and directions for future research.

 Regarding the process, weekly meetings were generally structured around case analysis or the sharing of experiences, with the role of the supervisor shifting from a “curious enquirer” or mediator to a more mentoring and ultimately facilitating role. The intervention was found to function in three distinct phases. While there was significant overlap between them, it can be summarized that the first phase required the express focus on building trust and rapport, emphasizing strengths in participants, in order to tackle the insecurities and interpersonal conflict arising from the experienced lack of competence. With some baseline of trust established, the second phase focused more on building competences through sharing experiences, which also had a significant effect on the participants’ perceived sense of work-related meaning and impact. Finally, in the third phase, true self-determination or autonomy was established, as the supervisees took the initiative in identifying areas of competence to work on together. By constant monitoring and adjustment on the part of the supervisor, the ESG was able to gradually move towards the empowered state necessary for mental health staff to make independent evaluations of complex situations and decide on a course of action, where improvements in competence, self-determination, and perceived meaning and impact as related to the empowerment model provided a useful unit of analysis. Within these phases, a gradual acclimatization to the online setting can also be seen, where the initial concerns about privacy, difficulty finding time and space and connection issues were resolved or became close to irrelevant.

 The first research question was what the process of the ESG would look like within the context of supervision of LMHW and junior psychologists in rural India. Though existing research has established the potential for clinical supervision at a distance, if not specifically supervision in a task-shared environment, the particular focus on empowerment in relation to complexity is entirely novel.^13,35-38^ Generally, multi-disciplinary supervision approaches have been found to be uniquely suited to enabling growth in handling complexity in clinical settings, which was similarly found to be the case in this study.^39^ Though more publications regarding supervision online may be forthcoming given the alterations to working environments arising from the COVID-19 pandemic, it should be considered whether the potential benefits (cost-effectiveness, less travel challenges) of digital supervision in low resource settings are generally considered with sufficient nuance regarding the degree of independent action that classical models actually enable.^33,40,41^ To that end, this study provides an example where the dynamic supervision strategy focused on participant needs through an empowerment framework might provide an example. Once consistency was established, the hour-long weekly supervision began to set concrete agendas and learning goals. This included, among other topics, legal and ethical issues, consultation on individual cases, client safety and participants’ development. Indeed, the importance of structure and consistency in clinical supervision has been highlighted in previous research in relation to the development of the professionals being.^42-44^ The three distinct phases in the nature of the supervision can also be linked to best practices in supervision, such as the need to build trusting relationships as a baseline.^44-46^ This approach does also highlight one of the challenges identified in literature on group supervision, where the advantages regarding resource intensity, peer learning and empathy which were also seen in this study are balanced by the demands on the supervisor.^47,48^ According to the literature settings require a supervisor who is able to understand both organizational issues and group dynamics and respond accordingly.^47,48^ This is in addition to the essential skills related to empowerment in particular, where both similarities and differences can be identified in how supervision links to empowering leadership.^27^ For instance, the context specific tools and moments of essential *in*action occurred in supervision as well, though the transition to a facilitating role is more specific to leadership practices.^27^

 The particular emphasis on managing power dynamics inherent in hierarchical structures, and the related issues with authority may be more characteristic of the particular study context regarding community clinics in India. Having established a positive learning environment, the second phase focused more on assessing participants’ strengths and providing feedback. This aspect is strongly characteristic of an empowerment-based approach as noted in systematic reviews, and may also be linked to the “restorative” function of supervision regarding its impact on well-being.^29,44,49,51^ With the associated improvement in confidence generated by this approach, it became possible for the final phase to pass more agency to the participants as they determined their own needs regarding administrative responsibilities, time management and team building. Though all aspects of empowerment played some role in each of the phases, this study contributes to knowledge on empowering leadership and supervision by building on the beginning qualitative understandings such as those of Bunders et al, relating to prioritization of different aspects of empowerment during different phases of the process, where for instance the fear of being found incompetent must be resolved before self-determination or understanding of impact and meaning can be considered.^27,30,52^

###  Supervisee Experiences

 The second research question was to identify the challenges participants experienced. As noted in relation to the process, the nature of the challenges and the supervisors’ tactics shifted in each of the different phases. For instance, in Phase 1, where practical challenges relating to bandwidth and connectivity occurred, here as in other literature the relationship between competence and fears of incompetence added a dimension to connectivity issues that related to interpersonal conflict.^53,54^ Indeed, such conflict in professional relationships resulting from a negative emotional load in care practitioners has also been identified elsewhere.^54^ With these conflicts addressed through building trust and planning of the first phase, the potential of tele-supervision relating to convenience and lack of geographical barriers became more visible.^19,55,56^ Yet the interplay of challenges related to (dis)empowerment continued across the phases in shifting forms. For instance, perceived incompetence had caused feelings of powerlessness for some participants that kept them from making their own decisions and thus from feeling their impact and meaning within the organization. This prerequisite faith in one’s own abilities is noted in other studies; as is the importance of participants and supervisor feedback and reassurance in addressing the issue.^44,57,58^ By reframing challenges as opportunities, participants were able to take decisive action that fostered their self-efficacy and drew them on into the third phase in which they themselves were able to set the agenda of the sessions and reach agreements together. The importance of this level of empowerment is further supported by the work of Kane et al which established the link between the ability of lay health workers to empower populations, and their own state of empowerment and abilities. Hence the rising trend of empowerment leadership, and the working definition of empowerment applied to lay health workers in numerous other studies also finds further support in this study.^28,59^

###  Use of Technology

 In spite of the discomfort experienced with the online medium in the first phase, it was gradually apparent that the use of technology ultimately contributed to participants’ autonomy in that they were able to join from their own working environments. This is especially significant giving the geographical distinctions in the different clinics of MHAT. This contributes to a growing body of literature on the promotion of ownership, equity and agency through online learning.^60-62^ Creating competence with online platforms, for instance using the whiteboard function of zoom, or the thumbs-up function to provide positive feedback was both a challenge and ultimately an impactful opportunity. Where initial concerns about privacy existed, the ability to watch back recordings and create joint note taking was ultimately experienced as a valuable extension of more classical supervisory formats.

###  Limitations

 There are limitations of this study, which include: (*a*) the use of purposive sampling; (*b*) data from a single organization; (*c*) small sample size; (*d*) potential response bias; and (*e*) varying levels of experience with peer supervision in respondents. Purposive sampling is a non-probability sampling technique that reduces the generalizability of the research findings and increases the likelihood of selection bias.^63^ The small sample size could also limit the generalizability of these research findings to the specific population.^64^ Regarding internal validity and sample size, it is not known whether these research findings can account for the full field and variation of the phenomenon being examined.^65^ In addition to the limitations associated with a small sample size, it is possible that some participants exhibited response bias when answering the interview questions. For instance, participants might respond in a way that is perceived as more socially desirable to the researcher.^66^ Other variables relating to the researcher’s demographic and interview characteristics could also potentially have biased participants’ responses. Variables such as the researcher’s role in the organization could significantly affect bias in participant response.^67^ Finally, a variation in the amount of supervision received across the sample as well as the level of experience of working was suggested as influencing LMHWs’ perspective and response time to interview questions. Experience in community clinics could significantly influence LMHWs’ perceptions regarding the extent of the role supervision played in the initial development of clinical skill sets.

## Conclusion

 This study explored the processual functioning of ESG in the context of junior psychologists and trained LMHWs in rural India, in order to determine the feasibility of empowering health workers to address the complexity of mental health through online supervision. The importance of empowerment through supervision and relating to complexity in particular is well established, yet the practical means of doing so in low resource and geographically restricted areas are still limited.^29,67^ The use of task-sharing to address human resource shortages has been established to involve a reconfiguring of specialists as supervisors and mentors of junior colleagues and LMHWs.^13,68^ This study found that it was possible to provide empowering supervision online through the application of dynamic supervision that focused on those aspects of empowerment that could be identified as the priority, resulting in a responsiveness to needs and improved outcomes.

###  Implications for Future Research

 In addition to a general call to consider the importance of supervision in task-shared environments and the potential of empowerment as a focus for such supervision, this research points to particular future directions regarding complex leadership. Future research should consider the question of the scalability of flexible and dynamic supervisory practices to trace the potential of replicating this work in other contexts. Furthermore, pursuing direct links between empowering supervision, complexity leadership and community outcomes could present interesting data. The relationship between different aspects of empowerment and the building of supervisory relationships over time would benefit from further research to establish trends in terms of priority-setting at different points in time. Such research should also consider the differential factors of different supervisors, as well as the level of homogeneity of experience and background of the participants. Quantifying the beneficial outcomes of empowerment-based approaches of supervision and training might also provide further impetus for the consideration of this method.

## Ethical issues

 Ethical approval for the study was granted by the Institute Clinical Research Ethics Committee of the MHAT, Calicut. Informed consent was obtained from the participants through an information leaflet. Data was collected between January and April 2021 through individual interviews. Participants could opt for telephone interviews in view of work schedules, location, and pandemic-related social distancing. All interviews were audio-recorded and conducted by experienced researchers who were not known to the participants and translated from Malayalam to English. Data was handled and stored according to MHAT’s Data Protection Policy, ensuring confidentiality.

## Competing interests

 Authors declare that they have no competing interests.
